# Oxidative Stress Biomarkers in Some Rat Brain Structures and Peripheral Organs Underwent Cocaine

**DOI:** 10.1007/s12640-012-9335-6

**Published:** 2012-07-12

**Authors:** Lucyna Pomierny-Chamioło, Andrzej Moniczewski, Karolina Wydra, Agata Suder, Małgorzata Filip

**Affiliations:** 1 Department of Toxicology, Faculty of Pharmacy, Medical College, Jagiellonian University, Medyczna 9, 30-688 Kraków, Poland; 2 Laboratory of Drug Addiction Pharmacology, Department of Pharmacology, Institute of Pharmacology, Polish Academy of Sciences, Smętna 12, 31-343 Kraków, Poland

**Keywords:** Rat, Oxidative stress, Cocaine self-administration, Superoxide dismutase, Malondialdehyde

## Abstract

Oxidative stress (OS) generates or intensifies cocaine-evoked toxicity in the brain and peripheral organs. The aim of this study was to examine superoxide dismutase (SOD) activity and lipid peroxidation [measured by malondialdehyde (MDA) levels] in rats during maintenance of cocaine self-administration and after withdrawal by a yoked-triad procedure. Our results indicate that repeated cocaine self-administration provoked an elevation of SOD activity in the hippocampus, frontal cortex, dorsal striatum, and liver. MDA levels were reduced in the brain, increased in the liver, kidney, and heart during maintenance of self-administration, and increased in the kidney in cocaine-yoked rats. In addition, following extinction training, we found enhanced MDA levels and SOD activity in the rat hippocampus, while changes in the activity of OS biomarkers in other brain structures and peripheral tissues were reminiscent of the changes seen during cocaine self-administration. These findings highlight the association between OS biomarkers in motivational processes related to voluntary cocaine intake in rats. OS participates in memory and learning impairments that could be involved in drug toxicity and addiction mechanisms. Therefore, further studies are necessary to address protective mechanisms against cocaine-induced brain and peripheral tissue damage.

## Introduction

Oxidative stress (OS) is defined as a disruption of redox signaling and control that can evoke malfunction in organs such as the brain (Dietrich et al. 2005; Bashkatova et al. 2006; Muriach et al. 2010; Macedo et al. 2010), heart (Devi and Chan 1999; Moritz et al. 2003a, b; Fineschi et al. 2001; Fan et al. 2009), liver (Devi and Chan 1996; Diez-Fernandez et al. 1999; Boelsteri and Goldlin 1991), kidney (Bemanian et al. 2005), spleen (Pacifici et al. 2003) or skin (Portugal-Cohen et al. 2010). OS gives rise to lipid peroxidation, protein oxidation, DNA damage, and several enzyme dysfunctions due to increase in the levels of reactive oxygen species (ROS).

Cocaine is an illegal and often abused psychostimulant, and the chronic consumption of cocaine causes damage in a range of body organs. Numerous different mechanisms of cocaine-evoked toxicity have been suggested, one of which is the intensification of OS. As found in vitro models, acute cocaine exposure in human neuronal progenitor cells causes increases in some OS biomarkers (Poon et al. 2007). In vivo studies, both acute and chronic passive cocaine treatments, evoked a significant increase in ROS production (e.g., H_2_O_2_), elevation in superoxide dismutase (SOD), glutathione peroxidase, and catalase activity in the rat cortex and striatum (Dietrich et al. 2005; Macedo et al. 2005). However, changes in brain catalase activity were not confirmed in the cortex and striatum in all studies (Macedo et al. 2005). In the hippocampus, but not in the frontal cortex, glutathione concentration and glutathione peroxidase activity were reduced in rats that were repeatedly treated with cocaine (Muriach et al. 2010). Cocaine intoxication also led to an increase in lipid peroxidation in several brain structures linked to dopamine synthesis and release (Dietrich et al. 2005; Bashkatova et al. 2006). Other studies concentrated on peripheral cellular enzyme and non-enzyme antioxidant defense systems and revealed that acute, passive cocaine administration resulted in an increase in ascorbic acid in the spleens of rats, while levels of glutathione and glutathione reductase were reduced (Pacifici et al. 2003). Moreover, the latter authors reported that chronic, passive cocaine treatment elevated glutathione, malondialdehyde (MDA), glutathione reductase, glutathione peroxidase, and SOD levels in the spleen along with a simultaneous depletion of ascorbic acid. Despite mentioned changes in OS biomarkers, literature data indicate that neither acute nor chronic cocaine administration was found to induce apoptosis in rat brain structures estimated by caspase-3 activity and TUNEL labeling (Dietrich et al. 2005; Muriach et al. 2010).

Investigations of OS biomarkers in rat brain structures following repeated cocaine treatment in vivo models are limited (see above), and the evaluation of active cocaine exposure with self-administration procedures has not yet been studied. Therefore, despite an existing body of knowledge regarding ROS generation after cocaine exposure, the possible role of ROS in cocaine addiction is far from clear. In the present study, self-administration procedures were employed to evaluate changes in SOD activity and MDA levels in the brain structures (hippocampus, dorsal striatum, and frontal cortex) and peripheral organs (liver, kidney, and heart) in cocaine addiction. Moreover, an extinction model was used in the self-administration procedures because it seems to be the most relevant animal model for studying the phenomenon of craving. We also employed a triad-yoked procedure in which each animal was paired with two rats that served as “yoked” controls; one rat received an injection of saline each time the paired rat self-administered a response-contingent injection of cocaine and the second rat received an injection of cocaine in the same way. The yoked procedure allowed us to distinguish between the pharmacological and motivational effects of psychostimulant intake.

## Materials and Methods

### Animals

The experiment was carried out on male Wistar rats (280–300 g) delivered by the licensed breeders (Charles River, Germany). Animals were housed individually in standard rodent cages in a colony room at a room temperature of 20 ± 1 °C and at 40–50 % humidity under 12-h light–dark cycle (lights on at 8:00). All experiments were conducted during the light phase of the light–dark cycle. Animals had free access to water and standard animal food during the 7-day habituation period. Rats were then maintained on limited water during the initial training session.

All the experimental procedures were carried out in accordance with the National Institutes of Health Guide for the Care and Use of Laboratory Animals and with approval of the Animal Care and Use Committee at the Institute of Pharmacology, Polish Academy of Sciences in Kraków.

### Drug

Cocaine hydrochloride (Sigma-Aldrich, USA) was dissolved in sterile 0.9 % NaCl and given iv (0.05 ml/infusion).

### Behavioral Procedures

#### Surgery

After 7-day habituation period, animals were water deprived for 18 h and then 2 days trained to press the lever for water reinforcement under a fixed ratio (FR) 1 schedule of reinforcement. On the third day of training, the number of responses required to produce reinforcement was increased to a final value of five. During this phase of training, the amount of water was restricted to that given during daily training sessions and after sessions for 10 min. Two days following lever-press training and free access to water, the rats were anesthetized with ketamine HCl (75 mg/kg, Bioketan; Biowet, Puławy, Poland) and Xylazine (5 mg/kg, Sedazin; Biowet, Puławy, Poland) and chronically implanted with a silastic catheter in the external jugular vein, as described previously (Filip et al. 2006). The catheters were flushed every day with 0.1 ml of saline solution containing heparin (70 U/ml, Biochemie GmbH, Austria) and 0.1 ml of solution of cefazolin (10 mg/ml; Biochemie BmbH, Austria). Catheter potency was tested periodically, or whenever an animal displayed behavioral outside baseline parameters, with the ultrashort-acting barbiturate anesthetic methohexital (10 mg/kg, iv) for loss of consciousness within 5 s.

#### Apparatus

Cocaine self-administration experiments were conducted in standard operant chambers (Med-Associated, St. Albans, USA) enclosed in ventilated, sound attenuating cubicles and controlled by an IBM-compatible computer by means of the MED Associates MED-PC software package. Each chamber was equipped with a 24-V house light, two retractable levers on the wall, a water-filled dispenser, a white circular stimulus light illuminated by a 24-V bulb above each lever, and a tone generator. Pressing on “active” lever resulted in drug delivery to the animal when scheduled (FR 5) requirements were met, whereas presses on the “inactive” lever were recorded but not reinforced.

### Self-Administration Procedures

Animals were divided into six separate groups (experimental design shown in Table 1); each of them consisted of 8 rats.

**Table 1:** Experimental design

Maintenance 16 days	Extinction 10 days	Group
‘Yoked’ saline (saline 0.1 ml/infusion)	–	1a
‘Yoked’ cocaine (cocaine 0.5 mg/kg/infusion)	–	1b
Cocaine self-administration (cocaine 0.5 mg/kg/infusion)	–	1c
‘Yoked’ saline (saline 0.1 ml/infusion)	Saline iv	2a
‘Yoked’ cocaine (cocaine 0.5 mg/kg/infusion)	Saline iv	2b
Cocaine self-administration (cocaine 0.5 mg/kg/infusion)	Saline iv	2c

After a 10-day recovery period, all animals were again water restricted and trained to press lever according to a FR 5 schedule of water reinforcement over a 2-h session. Then, subjects began lever pressing for cocaine reinforcement during 2-h daily sessions performed 6 days/week. The house light was illuminated throughout each session. Each completion of five press on the “active” lever complex resulted in a 5-s infusion of cocaine (0.5 mg/kg per 0.1 ml) and 5-s presentation of a stimulus complex, consisting of activation of the white stimulus light directly above the “active” lever and from a generator (2,000 Hz; 15 dB above ambient noise levels). Following each injection, there was a 20-s time-out period during which responding was recorded but had no programmed consequences. Response on the “inactive” lever not resulted in cocaine delivery. Acquisition of the conditioned operant response lasted a minimum of 10 days until subjects met the following criteria: minimum requirement of 22 reinforcements with an average of 6 days and active lever presses with an average of 6 consecutive days and a deviation within those 6 days of <10 % of the average; this criterion was selected based on our prior experiments (Fijał et al. 2010). During maintenance, rats administrated an average of 15–18 mg/kg cocaine (iv) across the 2-h session.

Following stabilization of responding, the extinction procedure was carried out. Subjects had 2-h daily training sessions with no delivery of cocaine and no presentation of the conditioned stimulus. Once they reached the extinction criteria (a minimum 10 extinction days with the responding on the active lever below 10 % of the level observed during at least three consecutive maintenance days).

After both maintenance and extinction phase of procedure animals were decapitated, brains and peripheral organs (liver, heart, and kidney) were removed. The frontal cortex, hippocampus, dorsal striatum, and organs from each animal were obtained by dissection, immediately frozen on dry ice, and stored at −80 °C.

### Neurochemical Procedures

#### SOD Activity

SOD activity in brain structures and peripheral tissues was assessed by the Misra and Fridovich method based on the ability of the enzyme (SOD) to inhibit the autoxidation of epinephrine (Misra and Fridovich 1972). The tissue was prepared immediately before analysis by homogenization in 10 volumes of double distilled cold water (4 °C) and then cleared by centrifugation at 20,000 rpm for 10 min at 4 °C. The supernatant was used for the enzyme assay. Analyses were performed in triplicate and the average values were taken. Protein content was measured by the Lowry method (Lowry et al. 1951) to take into account the protein content of each sample when expressing the biochemical results. Results were calculated as U/mg protein and on figures they are presented as % of corresponding control.

#### MDA Level

The extent of lipid oxidation was determined by measuring MDA levels. MDA is a low-weight, lipid peroxidation product that originates from the decomposition of highly reactive lipid hydroperoxides (Cherubini et al. 2005; Spickett et al. 2010). MDA levels in brain structures and peripheral tissues were estimated by the thiobarbituric acid (TBA) method. Samples were homogenized in 10 volumes of cold water. A MDA standard was prepared by hydrolysis of 16.4 μl of 1,1,3,3-tetraethoxypropane stock solution in 50 ml of 0.2 mM hydrochloric acid and incubated at 100 °C for 1 h. The MDA standard (10 mM) was further diluted to yield final concentrations of 1, 2, 3, 5, 7, and 10 μM to obtain the standard curve for estimation of total MDA. MDA levels in examined tissues were measured as follows: (1) 1 ml samples of homogenate were incubated with 1 ml of 0.37 % TBA in 50 mM NaOH and 1 ml of 2.8 % trichloroacetic acid in a boiling water bath for 20 min to develop a colored MDA–TBA adduct (TBA acid reactive species), and were then clarified by centrifugation at 1,500 rpm for 10 min, and 2); the resulting supernatants were aspirated, and the pink chromogen was measured at 532 nm using a Varian Cary 50 UV–Visible Spectrophotometer against a blank by comparison with the standard curve. The results were evaluated from the standard curve and calculated as μM MDA/g of tissue. All the analyses were performed in triplicate, and the average values were taken.

### Data Analysis

Data are presented as the mean ± SEM. In the cocaine self-administration experiment, the data were analyzed by Student *t* test (number of active and passive lever presses, infusions). In the biochemical assays, one-way analysis of variance (ANOVA) followed by post hoc Newman–Keuls tests were applied to evaluate statistically significant differences between the treatment groups. In addition, to separate the effects of treatment (saline vs. cocaine) and drug administration (self vs. yoked), we used the Student’s *t* test. The criterion for statistically significant differences was set at *p* < 0.05.

## Results

### Behavioral Studies

**Fig. 1:**
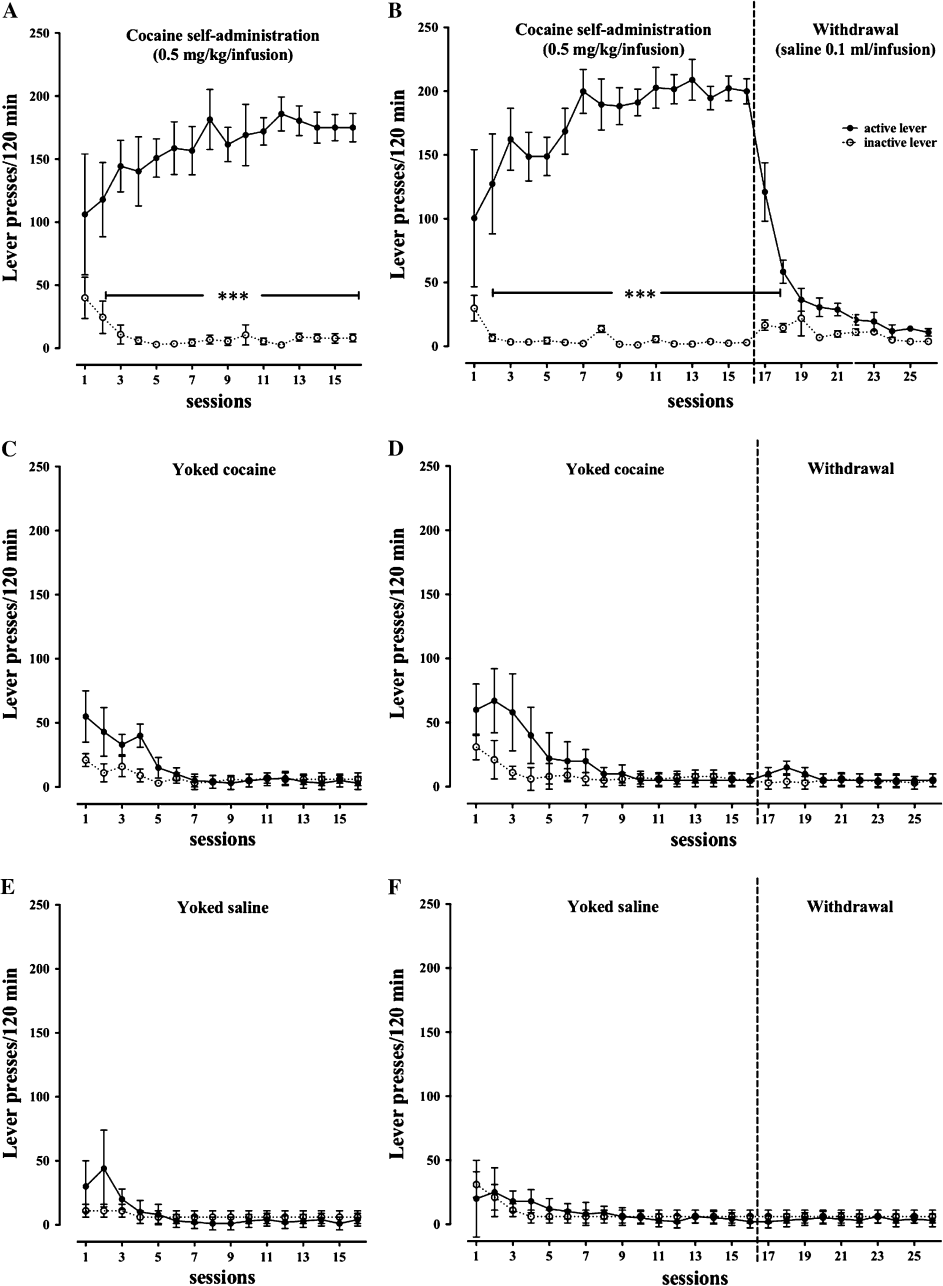
The mean number (±SEM) of responses in active and inactive levers for rats that acquired self-administration of cocaine at a dose of 0.5 mg/kg/injection and their yoked controls that received passive infusions of cocaine or saline. *p < 0.001 versus inactive lever. *N* = 6–8 rats/group

After 14 sessions of self-administration, the rats showed stable responding on levers during the last 6 self-administration maintenance sessions determined by an acquisition criterion requiring that the rate of active lever presses varied by less than 10 %. The rats responded significantly more frequently on the active lever than on the inactive lever (*p* < 0.01), independent of self-administration test day (Fig. 1a, b). The rats self-administered 24–35 injections of cocaine with a daily mean cocaine intake between 15 and 18 mg/kg (total mean cocaine intake during the 14 sessions was approximately 242 mg/kg/rat).

In the yoked cocaine group (Fig. 1c, d), the difference between responding on the active versus the inactive lever failed to reach significance. These animals passively received exactly the same amount of cocaine (daily mean cocaine intake between 15 and 18 mg/kg, total mean cocaine intake during the 14 sessions was approximately 242 mg/kg/rat) at the same time as the rats that learned to actively self-inject cocaine.

In the yoked saline group (Fig. 1e, f), the difference between responding on the active versus the inactive lever failed to reach significance.

### Biochemical Studies

#### Cocaine Self-Administration

##### Effects in the Brain

**Fig. 2:**
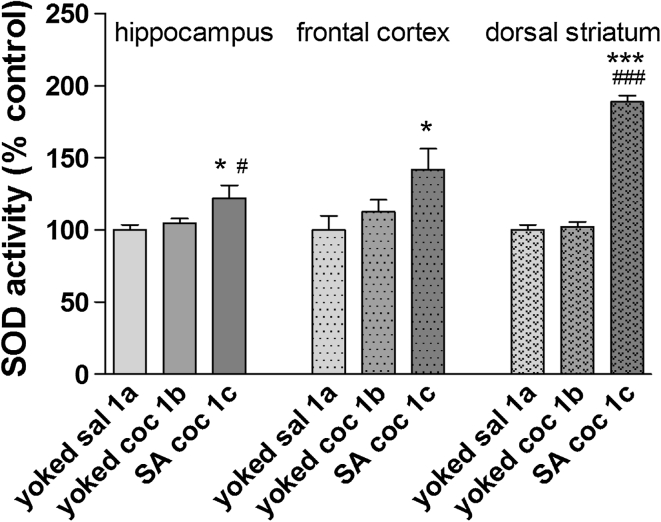
SOD activity in brain structures during cocaine (coc) self-administration (SA) by yoked-triad procedure. Data shown as a % of control ± SEM; *p < 0.05, ***p < 0.0001 versus yoked saline (sal); ^#^ *p* < 0.05, ^###^ *p* < 0.001 versus yoked cocaine. In control (yoked saline) rats, the absolute SOD activity was 7.64 ± 0.28 U/mg protein in the hippocampus, 4.03 ± 0.39 U/mg protein in the frontal cortex and 6.32 ± 0.48 U/mg protein in the dorsal striatum. *N* = 6–8 rats/group

The effects of active cocaine intake and passive cocaine administration on SOD activity in the rat hippocampus, frontal cortex, and dorsal striatum are shown in Fig. 2. Significant increases were found in the SOD activity in the hippocampus (+18 % ± 8 of control), frontal cortex (+41 % ± 14.5 of control, *p* < 0.05), and dorsal striatum (+88 % ± 9 of control, *p* < 0.001) after cocaine self-administration (group 1c) in comparison to the saline control group (group 1a).

**Fig. 3:**
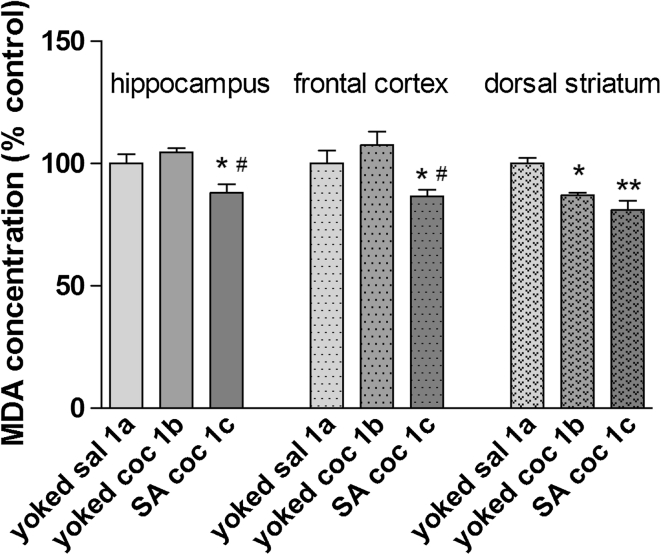
MDA concentration in brain structures during cocaine (coc) self-administration (SA) by yoked-triad procedure. Data shown as a % of control ± SEM; *p < 0.05, **p < 0.01 versus yoked saline (sal); ^#^ *p* < 0.05 versus yoked cocaine. In control (yoked saline) group, the absolute MDA level was 173 ± 6.4 μM/g tissue in the hippocampus, 143 ± 7.3 μM/g tissue in the frontal cortex and 179.6 ± 12.5 μM/g tissue in the dorsal striatum. *N* = 6–8 rats/group

A significant reduction in MDA levels was noted in the hippocampus (approximately −12 % of control) and frontal cortex (approximately −14 % of control, *p* < 0.05) in the self-administering cocaine group (Fig. 3). In the dorsal striatum, reductions of MDA levels in both the active (−16 % ± 7, *p* < 0.05) and passive (−11 % ± 0.5, *p* < 0.01) groups were observed.

##### Effects in Peripheral Organs

**Fig. 4:**
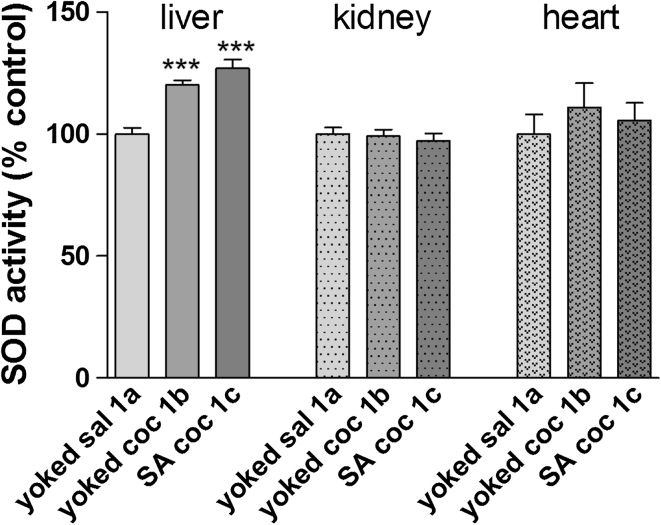
SOD activity in peripheral organs during cocaine (coc) self-administration (SA) by yoked-triad procedure. Data are shown as mean ± SEM; ***p < 0.001 versus yoked saline (sal). In control (yoked saline) rats, the absolute SOD activity was 3.5 ± 0.09 U/mg protein in the liver, 7.3 ± 0.19 U/mg protein in kidney and 6.4 ± 0.52 U/mg protein in the heart. *N* = 6–8 rats/group

As illustrated in Fig. 4, compared to the yoked saline control, chronic exposure to cocaine enhanced the SOD activity in the liver of animals that actively (+26 % ± 3.6, *p* < 0.001) and passively (+20 % ± 1.8) administered cocaine. In the kidney and heart tissues, no statistically significant alterations were seen.

**Fig. 5:**
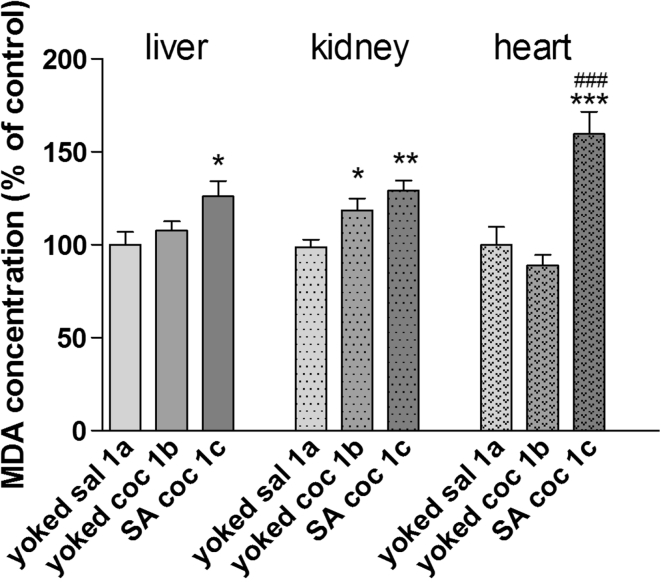
MDA concentration in peripheral organs during cocaine (coc) self-administration (SA) by yoked-triad procedure. Data are shown as mean ± SEM; *p < 0.05, **p < 0.01, ***p < 0.001 versus yoked saline (sal); ^###^ *p* < 0.001 versus yoked cocaine (coc). In control (yoked saline) group, the absolute MDA level was 20.67 ± 1.4 μM/g tissue in the liver, 328.5 ± 13.42 μM/g tissue in kidney and 17.72 ± 1.6 μM/g tissue in the heart. *N* = 6–8 rats/group

During the maintenance phase, MDA levels in the heart and the liver were comparable and were, respectively, 17.72 ± 1.6 and 20.67 ± 1.4 μM/g in the control groups. The kidney MDA concentration was 15- to 18-fold higher than in other tissues. We observed a significant increase in the amount of MDA in all analyzed organs (liver *p* < 0.05, kidney *p* < 0.01, and heart *p* < 0.001) after active cocaine administration and in the kidney from animals that were administered cocaine passively (*p* < 0.05; Fig. 5).

#### Extinction Training

##### Effects in the Brain

**Fig. 6:**
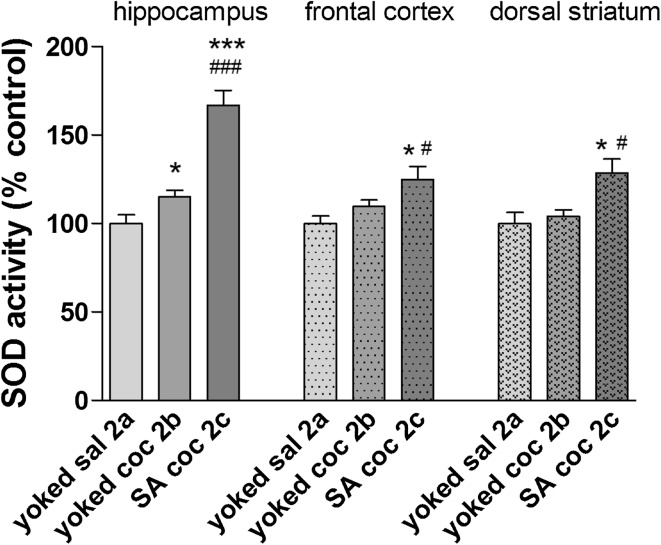
SOD activity in brain structures after cocaine (coc) self-administration (SA) and extinction training by yoked-triad procedure. Data shown as a % of control ± SEM; *p < 0.05, ***p < 0.001 versus yoked saline (sal); ^#^ *p* < 0.05, ^###^ *p* < 0.001 versus yoked cocaine. In control (yoked saline) rats, the absolute SOD activity was 6.3 ± 0.3 U/mg protein in the hippocampus, 3.92 ± 0.36 U/mg protein in the frontal cortex and 5.36 ± 0.62 U/mg protein in the dorsal striatum. *N* = 6–8 rats/group

As shown in Fig. 6, the SOD activity in the rat hippocampus significantly increased on extinction day 10 following earlier active (+66 % ± 8.5 of control, *p* < 0.001) and passive (+15 % ± 4 of control, *p* < 0.05) injections of cocaine. In the rat frontal cortex and dorsal striatum, only extinction from active cocaine administration significantly enhanced the level of SOD activity.

**Fig. 7:**
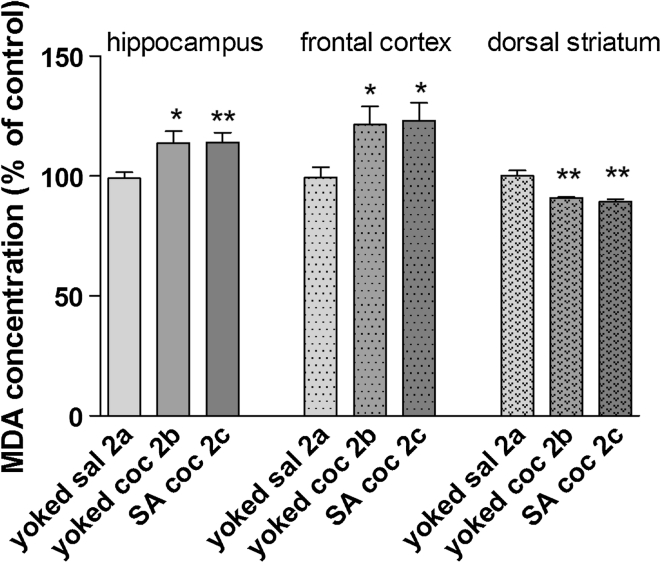
MDA concentration in brain structures after cocaine (coc) self-administration (SA) and extinction training by yoked-triad procedure. Data shown as a % of control ± SEM; *p < 0.05, **p < 0.01 versus yoked saline (sal). In control (yoked saline) group, the absolute MDA level was 182.4 ± 5.6 μM/g tissue in the hippocampus, 142 ± 7.8 μM/g tissue in the frontal cortex and 186 ± 11.9 μM/g tissue in the dorsal striatum. *N* = 6–8 rats/group

The changes in MDA concentrations in brain structures are shown in Fig. 7. The MDA levels were significantly elevated in the rat hippocampus and frontal cortex after both active (hippocampus: +13 % ± 1.2 of control, *p* < 0.05; frontal cortex: +23 % ± 3.1 of control *p* < 0.05) and passive (hippocampus: +14 % ± 1.4 of control, *p* < 0.01; frontal cortex +22 % ± 3.3 of control) cocaine administration, while the MDA levels in the dorsal striatum were decreased in both groups (approximately 17 % of control, *p* < 0.01).

##### Effects in Peripheral Organs

**Fig. 8:**
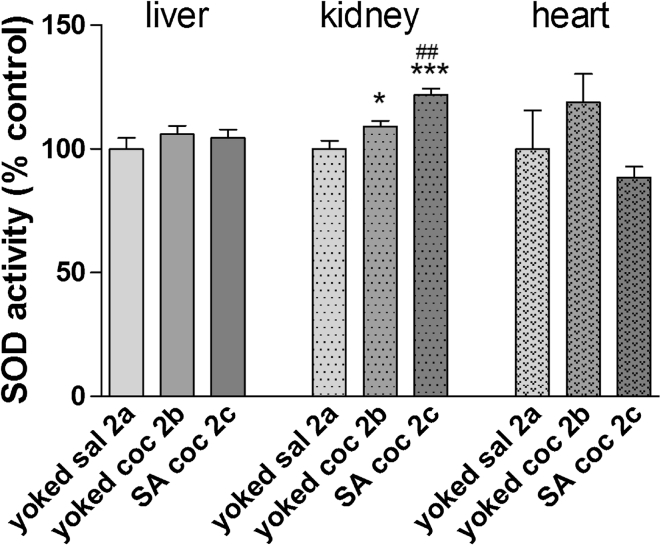
SOD activity in peripheral organs after cocaine (coc) self-administration (SA) and extinction training by yoked-triad procedure. Data shown as a % of control ± SEM; *p < 0.05, ***p < 0.001 versus yoked saline (sal); ^##^ *p* < 0.01 versus yoked cocaine. In control (yoked saline) rats, the absolute SOD activity was 3.2 ± 0.14 U/mg protein in the liver, 5.8 ± 0.19 U/mg protein in kidney and 5.4 ± 0.74 U/mg protein in the heart. *N* = 6–8 rats/group

Figure 8 shows the effects of cocaine withdrawal on SOD activity in peripheral organs in rats. A significant enhancement in SOD activity was seen in the kidney of animals receiving active (+21 % ± 2.5, *p* < 0.001) and passive (+12 % ± 2.1, *p* < 0.05) cocaine injections. No changes were reported in the liver or in the heart.

**Fig. 9:**
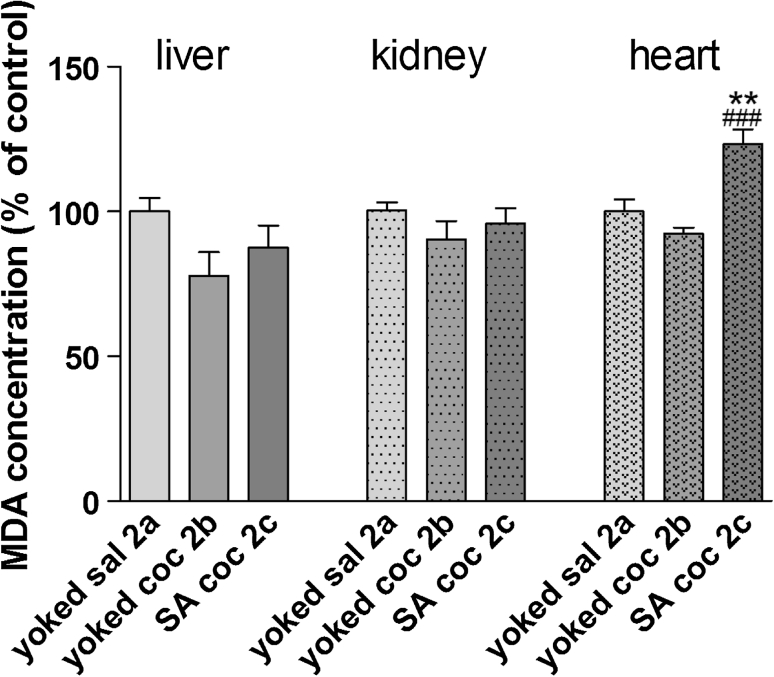
MDA concentration in peripheral organs after cocaine (coc) self-administration and extinction training by yoked-triad procedure. Data are shown as mean ± SEM; **p < 0.01 versus yoked saline (sal); ^###^ *p* < 0.001 versus yoked cocaine. In control (yoked saline) group, the absolute MDA level was 27.44 ± 2 μM/g tissue in the liver, 332 ± 10.17 μM/g tissue in kidney and 19.15 ± 1.0 μM/g tissue in the heart. *N* = 6–8 rats/group

In the liver, potent increase in the MDA levels were seen in animals during the maintenance of cocaine self-administration (+32 % ± 3.5 of control; *p* < 0.01), but in extinction phase were comparable in all groups (Fig. 9). The MDA levels in the heart of rats withdrawn from active cocaine injections were also significantly increased (+23 % ± 5 of control, *p* < 0.001). We observed no changes occurred in the kidney.

The control values in SOD activity and MDA levels are almost similar in yoked saline rats underwent cocaine self-administration and extinction training (see legends to Figs. 2, 3, 4, 5, 6, 7, 8, 9).

## Discussion

The present findings demonstrate that the motivational and pharmacological processes related to cocaine intake in rats result in differing levels of some OS biomarkers in the brain and peripheral tissues. Apart from the well-known peripheral toxicity induced by cocaine (Devi and Chan 1996; Fan et al. 2009; Bemanian et al. 2005), many brain structures such as the dorsal striatum, the frontal cortex, and the hippocampus have been studied in relation to drug-mediated acute intoxication, addiction-related neuroadaptations and/or memory functions (e.g., McClelland et al. 1995). The present study focused on concurrent ex vivo evaluation of lipid oxidation and antioxidant defense mechanisms and examined the amount of MDA formation, which is an end product of membrane fatty acid peroxidation (Halliwell and Gutteridge 1997), and the activity level of SOD, which is the enzyme that scavenges superoxide anions (Fridovich 1989).

Repeated cocaine exposure produced significant changes in the oxidative status in the rat. We found a significant increase in the MDA levels (up to 50 %) in the liver, kidney, and heart as well as a small (15–25 %) decrease in some limbic and subcortical brain areas. Interestingly, there was an enhancement of enzymatic SOD activity in the hippocampus, frontal cortex, dorsal striatum, and liver. The most intriguing finding was that self-administered cocaine evoked more potent oxidative damage and altered the antioxidant defense in rats (group 1c), while the same amount of cocaine administered passively in yoked animals (group 1b) did not change brain OS biomarkers with the exception of the striatal MDA level. Similar divergences between repeated active and passive cocaine injections were seen in peripheral tissues (liver and heart) where cocaine self-administration provoked membrane acid peroxidation.

In accordance with our present findings, several preclinical and clinical studies indicate renal toxicity in peripheral organs following acute or chronic cocaine intake (Kramer and Turner 1993; Saleem et al. 2001; Edmondson et al. 2004). Rhabdomyolysis, hypertension, microangiopathy, and glomerular ischemia are the most common pathogenetic factors in the kidney, but OS (Bemanian et al. 2005) and the generation of ROS (Kovacic et al. 2002) have been proposed to contribute in the development of renal infraction. We found increased MDA concentrations in renal tissues in both the active and passive cocaine groups, but the level of SOD activity remained unchanged. The latter enzyme catalyzes the conversion of the superoxide anion to hydrogen peroxide (Diez-Fernandez et al. 1999), which may indicate that the MDA levels in the kidney increased due to the formation of other ROS. A similar suggestion arises for the potent lipid peroxidation due to active cocaine injections observed in the rat heart. Cocaine use is a risk factor for myocardial infarction, coronary artery spasm, arrhythmia and ischemia (Devi and Chan 1999; Vongpatanasin et al. 1999), and the mechanisms linked to drug toxicity come from disturbances in OS biomarkers (higher MDA levels and SOD activity, lower glutathione level, impairment of catalase, and glutathione peroxidase activity in the myocardium (Devi and Chan 1999; Moritz et al. 2003a). The latter effects were seen following passive cocaine injections in rats (Devi and Chan 1999; Moritz et al. 2003a); however, the cocaine dose-range and the drug treatment time frame were much higher or longer, respectively, than in our study.

Interestingly, we report differences in OS status not only in rat peripheral tissues but also in the brain. In fact, only rats given active (voluntary) cocaine injections displayed an enhancement in SOD activity levels in the dorsal striatum, frontal cortex, and hippocampus despite the fact that both experimental cocaine groups (self-administered and yoked cocaine) were subjected to the same dose and schedule of cocaine administration. The increase in SOD activity has been varied (from 18 to 88 %). The highest activity has been reported in dorsal striatum. It should be added that increases in the SOD enzymatic activity corresponded to a reduction in MDA concentrations in all the investigated brain areas.

Another main result from the present study is that, in the drug-free conditions that followed extinction training, there were significant increases in MDA levels in rats previously exposed to cocaine (either active or passive) in the hippocampus (ca. 13 %) and frontal cortex (ca. 23 %). In conjunction with MDA levels, intensification of SOD activity was reported in the hippocampus (66 %) and kidney (21 %) in rats injected with cocaine, as well as in the frontal cortex (25 %) and dorsal striatum (30 %) in animals previously linked with active cocaine intake. These data suggest a correlation between the lipid peroxidation and SOD activity and pharmacological mechanisms linked to earlier cocaine intoxication. It should be noted that the cellular antioxidant system in the rat striatum was not overwhelmed by ROS as escalated lipid peroxidation was not observed after cocaine withdrawal. In fact, decreases in MDA levels were reported. These findings point to the resistance of some brain areas to ROS following cocaine intake, despite increased SOD activity (see above).

In concurrence with the present findings, previous reports showed increases in lipid peroxidation in the rat frontal cortex and striatum (Dietrich et al. 2005) as well as in the prefrontal cortex and nucleus accumbens (Numa et al. 2008) after withdrawal from passive cocaine administration (Dietrich et al. 2005). Bashkatova et al. (2006) described such increases in lipid peroxidation in the hippocampus and cortex in young animals exposed to cocaine in utero. It should be mentioned that, despite increased lipid peroxides (Dietrich et al. 2005) or decreased glutathione concentration and glutathione peroxidase activity (Muriach et al. 2010), there was no apoptosis in the rat brain structures following repeated passive i.p. cocaine administration. Whether iv cocaine produces neurodegeneration further studies with caspase-3-dependent apoptosis are necessary to perform.

The present results indicate the presence of OS both during cocaine intake and after withdrawal in rats. Importantly, brain structures linked to the rewarding effects of drugs of abuse, motivational processes, and the formation of maladaptative cellular changes are implicated in drug addiction processes. There are several proposed mechanisms that may occur during cocaine intoxication and change the oxidative status of organs; the most important of which is a massive increase in dopamine release. In fact, cocaine binds to the transporter sites for monoamines (dopamine, noradrenaline, and serotonin) (Koe 1976) that results in an inhibition of their uptake into presynaptic neurons (Ritz et al. 1987). Enhanced neurotransmitter levels, primarily of dopamine, in the synaptic cleft prompt increases in levels of ROS (Stokes et al. 1999; Nestler 2004). More specifically, dopamine is metabolized either non-enzymatically (via molecular oxygen through autoxidation) to generate hydrogen peroxide (H_2_O_2_) and a superoxide anion (O_2_^−^), or enzymatically by monoamine oxidase to form H_2_O_2_. Both of these ROS may react via the Haber–Weiss/Fenton reaction with transition metal ions to produce the highly toxic hydroxyl radical (*OH) (Hermida-Ameijeiras et al. 2004). It was shown that the amount of dopamine released in the rat striatum is much higher when the rats self-administer cocaine (due to the effects of motivation in drug-experienced animals) than during passive injection of the same amount of cocaine (Suto et al. 2010; Wydra et al. 2011). Elevated dopamine levels may escalate ROS generation leading to the induction of a compensatory mechanism, such as an increase in SOD activity.

Interestingly, discrepancies between the active and passive cocaine injections were reported for glutamatergic neurotransmission with elevation in subcortical glutamate levels during cocaine self-administration and depression below baseline during yoked cocaine administration (Suto et al. 2010). Exposing neurons to high glutamate concentrations results in the activation of NMDA and AMPA receptors, which induces faster mitochondrial Ca^2+^ uptake (Rego and Oliveira 2003). High Ca^2+^ levels lead to the activation of the permeability transition pore in brain mitochondria followed by the formation of mitochondrial ROS and lipid peroxidation (Maciel et al. 2001). Increased Ca^2+^ levels also activate several intracellular enzymes (e.g., phospholipase A_2_, nitric oxide synthase, calcineurin, endonucleases, and xanthine dehydrogenase), which by themselves can induce the formation of ROS (Rego and Oliveira 2003). In support of such a statement, repeated cocaine administration evokes NMDA receptor upregulation in humans, non-human primates, and rodents (Crespo et al. 2002; Hemby et al. 2005) that persists long after discontinuation of drug treatment. Moreover, the dopamine metabolite quinine, a product of the auto-oxidative pathway, acts not on dopamine receptors, but on NMDA glutamatergic receptors (Lieb et al. 1995) and was shown to be neurotoxic in striatal neurons (Ben-Shachar et al. 1995). This dopamine metabolite appears during cocaine-provoked changes in organ oxidative status and links the dopamine-glutamine interaction with drug-mediated toxicity (Smythies 1997).

Another recent theory indicates impaired glutamate homeostasis in the nucleus accumbens due to active cocaine intoxication resulting from the cocaine-induced down-regulation of the cystine–glutamate exchanger during cocaine withdrawal (Kalivas 2009). This exchanger plays an important role in intracellular cysteine transport for glutathione production, a major cellular antioxidant. It was reported that chronic exposure to cocaine in rats results in reductions in the level of glutathione and causes an imbalance between ROS and antioxidant concentrations (Lipton et al. 2003), as well as an increase in the protein S-glutathionylation and a decrease in expression of GSH-S-transferase (Uys et al. 2011). Further studies supporting the importance of glutamatergic neurotransmission in cocaine addiction show that glycine, which is a co-activator of NMDA receptors, enhances the excitotoxic events related to glutamatergic neurotransmission (Berger et al. 1998; Bergeron et al. 1998; Dubroqua et al. 2010) while inhibiting glycine transporter-1, facilitates cocaine-cue extinction, and attenuates reacquisition of cocaine-seeking behavior (Nic Dhonnchadha et al. 2011).

It should be remembered that oxidized cocaine metabolites are involved in drug-mediated tissue damage. In fact, cytochrome P450 and flavin-containing monooxygenases, which are oxidative metabolites of cocaine, appear in addition to the appearance of bioactive norcocaine and N-hydroxynorcocaine or norcocaine nitroxide (Boess et al. 2000). Further oxidative metabolism and redox cycling between these cocaine metabolites lead to NADPH depletion and the generation of H_2_O_2_, superoxide anion (O_2_^−^), and other ROS (Boelsteri and Goldlin 1991; Bouis and Boelsterli 1990; Kovacic 2005). This mechanism may be present in the organs examined in this study where almost identical levels of MDA or SOD activity were observed following active or passive cocaine intoxication. Moreover, similar changes in the oxidative status of both cocaine groups exclude a role for motivational processes in such alterations.

In summary, the present study indicated that repeated self-administration of cocaine provoked an elevation of SOD activity in some brain structures and the liver, while MDA levels were either reduced (brain) or increased (peripheral tissues). In addition, following extinction training, we found enhanced MDA levels and SOD activity in the rat hippocampus—a brain area related to memory (Berke and Eichenbaum 2001). A very recent literature data show that intracerebral injection of SOD inhibits long-term potentiation—a fundamental process that modulate synaptic transmission and play a crucial role in neural mechanisms of memory (Viggiano et al. 2011). Changes in the activity of OS biomarkers in other brain structures and peripheral tissues were reminiscent of the changes seen during the self-administration of cocaine.

Our results highlight, for the first time, an association between OS biomarkers in the motivational processes related to voluntary cocaine intake in rats. OS plays a role in memory and learning, and impairments in these functions could be involved in drug toxicity and addiction mechanisms. Further studies are necessary to determine the protective mechanisms that inhibit cocaine-induced damage to the brain and peripheral tissues.

## Abbreviations

SOD: Superoxide dismutase
OS: Oxidative stress
MDA: Malondialdehyde
ROS: Reactive oxygen species
FR: Fixed ratio
TBA: Thiobarbituric acid
